# Cluster analysis to define distinct clinical phenotypes among septic patients with bloodstream infections

**DOI:** 10.1097/MD.0000000000015276

**Published:** 2019-04-19

**Authors:** Maria Cristina Vazquez Guilamet, Michael Bernauer, Scott T. Micek, Marin H. Kollef

**Affiliations:** a Division of Pulmonary, Critical Care, and Sleep Medicine; b Division of Infectious Diseases, University of New Mexico Health Sciences Center, Albuquerque, NM; c Division of Health Sciences Library and Informatics Center, University of New Mexico, Albuquerque, NM; d Department of Pharmacy Practice, St. Louis College of Pharmacy, St. Louis, MO; e Division of Pulmonary and Critical Care Medicine, Washington University School of Medicine, St. Louis, MO.

**Keywords:** bloodstream infection, machine learning, outcomes, sepsis

## Abstract

Prior attempts at identifying outcome determinants associated with bloodstream infection have employed a priori determined classification schemes based on readily identifiable microbiology, infection site, and patient characteristics. We hypothesized that even amongst this heterogeneous population, clinically relevant groupings can be described that transcend old a priori classifications.

We applied cluster analysis to variables from three domains: patient characteristics, acuity of illness/clinical presentation and infection characteristics. We validated our clusters based on both content validity and predictive validity.

Among 3715 patients with bloodstream infections from Barnes-Jewish Hospital (2008–2015), the most stable cluster arrangement occurred with the formation of 4 clusters. This clustering arrangement resulted in an approximately uniform distribution of the population: Cluster One “Surgical Outside Hospital Transfers” (21.5%), Cluster Two “Functional Immunocompromised Patients” (27.9%), Cluster Three “Women with Skin and Urinary Tract Infection” (28.7%) and Cluster Four “Acutely Sick Pneumonia” (21.8%). *Staphylococcus aureus* distributed primarily to Clusters Three (40%) and Four (25%), while nonfermenting Gram-negative bacteria grouped mainly in Clusters Two and Four (31% and 30%). More than half of the pneumonia cases occurred in Cluster Four. Clusters One and Two contained 33% and 31% respectively of the individuals receiving inappropriate antibiotic administration. Mortality was greatest for Cluster Four (33.8%, 27.4%, 19.2%, 44.6%; *P* < .001), while Cluster One patients were most likely to be discharged to a nursing home.

Our results support the potential for machine learning methods to identify homogenous groupings in infectious diseases that transcend old a priori classifications. These methods may allow new clinical phenotypes to be identified potentially improving the severity staging and development of new treatments for complex infectious diseases.

## Abstract 2

Supplemental Digital Content is available in the text

**Main Point**

- Cluster analysis applied to hospitalized septic patients with bloodstream infections identified 4 stable clusters correlating with clinical outcomes. Our results support the potential for machine learning methods to identify more homogenous infectious disease groupings that transcend old a priori classifications.

## 1 Introduction

Bloodstream infections (BSIs) represent the seventh leading cause of mortality with rates as high as 40% in most studies.^[1]^ Usually considered the consequence of a serious infection that arises elsewhere in the body and subsequently spreading to the bloodstream, bacteremia complicating primary infections has been shown to dramatically amplify the mortality associated with these infections.^[2,3]^ Moreover, increasing antimicrobial resistance especially among Gram-negative bacteria (GNB) has contributed to the complexity of treating these types of infection.^[4]^ Further limiting clinicians’ ability to objectively determine optimal antimicrobial treatment strategies for patients with BSI are the limited availability of clinically relevant profiles of such patients linked to clinical outcomes.

Recently, opposing results have been produced by 2 groups of investigators examining the relationship between the duration of antimicrobial treatment and clinical outcome among patients with *Enterobacteriaceae* BSI despite similar appearing patient populations and statistical methodologies.^[5–7]^ Such contradictory findings are relatively commonplace making generalizability difficult in regards to antimicrobial treatment decisions and signaling the likely heterogeneity that characterizes patients with similar infectious diseases. Prior attempts at trying to gauge the outcome determinants associated with serious infections have typically employed a priori determined classification schemes based on readily identifiable microbiology characteristics (causative agents of infection including GNB, *Staphylococcus aureus*, *Candida* spp), primary site of the infection (e.g., pneumonia, urinary tract, intra-abdominal), and patient characteristics (e.g., critically ill patients, bone marrow transplant recipients, trauma).^[8–11]^ Unfortunately, this type of approach for classifying patients fails to take into account the important interactions that likely occur among these characteristics.

Our objective was to explore the grouping of critically ill patients with bacteremia by reducing the multidimensionality of data while still preserving homogenous groups.

Cluster analysis is an unsupervised machine learning methodology that can discover more homogenous groups within heterogeneous sets of data.^[12]^ Cluster analysis has recently been employed to describe novel groupings of individuals within diverse disease states including chronic obstructive pulmonary disease, asthma, psychiatric disorders, and various malignancies.^[13–18]^ We hypothesized that even amongst the heterogeneous population of patients with BSIs, clinically relevant groupings can be described that transcend old a priori classifications. Improved ability to distinguish subgroups of infected patients for specific therapeutic strategies could lead to improved outcomes and potentially less emergence of antimicrobial resistance.

## 2 Methods

### 2.1 Setting and participants

This study was conducted at Barnes-Jewish Hospital in St Louis (1300 beds) and the Washington University School of Medicine Institutional Review Board waived informed consent. All adult patients with BSIs and severe sepsis or septic shock who were hospitalized between January 2008 and April 2015 were eligible for inclusion. BSI was defined as the presence of at least 1 positive blood culture with a true pathogen, or multiple positive cultures with a compatible clinical scenario in the case of isolating typical contaminant species (e.g., coagulase-negative staphylococci). We recorded all episodes of BSIs but only the initial episode for each patient was used in this analysis. Data were collected from the hospital's electronic medical record (EMR) provided by the Center for Clinical Excellence, BJC Healthcare. This data repository includes diagnoses, Charlson, and APACHE II scores, laboratory, microbiology, imaging results, and pharmacy records. Additionally, we manually checked the time frame for the presence of central venous catheters and mechanical ventilation. Infection source was determined based on concomitant positivity of sterile cultures (cerebral spinal fluid, pleural, bronchoalveolar lavage, tissue, joint aspirate) plus descriptive diagnoses in the EMR, when absent an unknown source of infection was assigned.

Previous antibiotics was defined as intravenous administration of antimicrobial agents within 30 days of the index episode of BSI, while previous hospitalizations had to occur within 90 days. Immunosuppression was defined as having the acquired immune deficiency syndrome, solid organ transplant, bone marrow/stem cell transplant, hematologic malignancies, solid cancers treated with chemotherapy or radiation, long term corticosteroid administration (greater than 2 weeks at greater than 10 mg/day of prednisone equivalent), and other immune suppressive drugs such as biologics for rheumatologic disorders. Septic shock was considered present when vasoactive agents (norepinephrine, epinephrine, vasopressin, phenylephrine) were used. EMR data for analysis was available for patient admissions to any of the fifteen BJC hospitals.

### 2.2 Microbiology and pharmacology methods

For our analyses, bacterial species were grouped into the following categories: *S aureus* (methicillin-susceptible and methicillin-resistant strains), *Streptococcus pneumoniae*, *Enterobacteriaceae*, non-fermenting GNB, *Candida* spp, anaerobes, other Gram-positive cocci including *Streptococcus* spp (not pneumoniae) and *Enterococcus* spp, and other GNB. Antimicrobial susceptibility testing was standardized and was determined using the Phoenix BD Automated System (BD Diagnostics, Sparks, Maryland).

From January 2002 through the present, Barnes-Jewish Hospital utilized an antibiotic control program during which time the use of intravenous ciprofloxacin, imipenem, meropenem, piperacillin/tazobactam, ceftolozone/tazobactam, ceftazidime/avibactam, linezolid, or ceftaroline was restricted and required preauthorization from an infectious diseases physician or clinical pharmacist. However, patients in the Intensive Care Unit setting could be empirically started on any antimicrobial regimen for the first 24 hours pending subsequent review. Appropriate antibiotic therapy was considered to be present based on subsequently documented *in vitro* activity of the empirically selected antimicrobial regimen against the isolated microbe(s) and had to be started within 24 hours of the positive blood cultures being drawn.

### 2.3 Statistical plan

Variables are reported as proportions, means and standard deviations or medians and interquartile range as appropriate.

#### 2.3.1 Feature selection

All collected variables were considered as potential candidate variables for cluster analysis and were selected from three domains: patient characteristics, acuity of illness/clinical presentation and infection characteristics. Patient characteristics included: age, gender, comorbidities, immunosuppression, prior hospitalization, prior exposure to intravenous antibiotics, recent surgery (abdominal versus non-abdominal), use of total parenteral nutrition, presence of a central vein catheter, admission source (home, nursing home, transfer from outside hospital), duration of hospitalization prior to the index BSI. Acuity of illness/clinical presentation features encompassed the need for vasopressors, use of mechanical ventilation, and APACHE II scores. Infection characteristics included the bacterial species, the source of infection, and the administration of appropriate antibiotic therapy. Non-normally distributed variables were log transformed. The initial iteration of the clustering analysis used all variables. We wanted to reduce the high dimensionality of data (3715 patients with more than 25 characteristics each) to obtain a parsimonious model that could be useful clinically. In subsequent iterations, variables that did not add to the robustness of the clustering algorithm that is, they were equally distributed among the clusters were dropped while checking the lack of change in the make up of the groupings.

#### 2.3.2 Consensus clustering

Cluster analysis refers to a broad set of unsupervised learning techniques used to discover distinct subgroups or clusters within a set of data. The goal of clustering is to partition observations into distinct groups in which observations assigned to the same group are similar with respect to one or more attributes while observations assigned into different groups are dissimilar. The process is unsupervised since it requires no a priori specification of group organization.

Consensus clustering is a clustering procedure that provides quantitative and visual evidence of cluster stability through repeated subsampling and clustering of the original data set.^[19]^ We specified a subsampling parameter of 80% with 1000 repetitions and the number of potential clusters (k) ranging from 2 to 9, in order to avoid producing an excessive number of clusters that would not be clinical useful. This also helps to provide stability in the setting of probable sampling variability. Binary variables were treated as being symmetrical. The selected clustering algorithm was the partitioning around medoids method.^[20]^ For each number of clusters, the algorithm calculates and retains the proportion of runs in which 2 observations are grouped together called pairwise consensus values. Due to the presence of mixed data (e.g., binary and continuous variables) we computed pairwise distances between each observation using Gower's distance.^[19]^ We assessed cluster stability by visually inspecting the diagnostic plots produced by ConsensClusterPlus including the consensus matrix and the cumulative distribution function plots. In addition, given the documented limitations of consensus clustering in choosing the number of clusters (k), we also computed the proportion of ambiguous clustering (PAC) to help select the most appropriate value for clinically relevant k. This represents the difference between pairs always clustered together and pairs never clustered together. The smallest PAC renders the optimal k.

#### 2.3.3 Cluster validity

We assessed cluster validity using multiple approaches. After performing the cluster analysis and choosing the most appropriate value for k, we compared each of the clusters and categorized them into distinct clinical phenotypes on the basis of their clinical characteristics (i.e., content validity). We compared outcome measures (discharge disposition and mortality) across each of the clusters (i.e., predictive validity). We hypothesized that valid, clinically distinct phenotypes would have measureable differences in outcomes. We assessed the stability of each cluster by inspecting the distribution of consensus values for each of the cluster members. Stable clusters typically have high mean consensus values with low variance. For each of the clusters, we then tabulated the total number of observations with consensus values 2 standard deviations less than the cluster mean, so called “outliers”. These observations represent admissions that were the least representative of the cluster and we then looked at “purified” cluster characteristics after removing these outliers. We performed consensus clustering using the ConsensClusterPlus package available in R project for statistical computing version 3.4.4.

We tried to limit selection bias by including all patients who had developed bacteremia during the study period. In order to avoid inaccuracies in electronic health records mining, after collection, data, and time stamps were manually verified.

## 3 Results

Three thousand seven hundred fifteen patients with BSIs and severe sepsis or septic shock met our inclusion criteria. The mean age was 58.4 ± 15.6 years and most patients were admitted from home (66.5%) (Table 1). More than one-third of our study population had immunosuppression and more than half of the study cohort had recently received intravenous antibiotics. The most common comorbidities were active cancer, diabetes, and chronic obstructive pulmonary disease. Septic shock was present in 45% of patients while 29.7% required mechanical ventilation. The most common sources of infection were pneumonia (27.7%) and urinary tract (22.0%). Inappropriate antibiotic therapy was administered to 25.4% of patients, while *Enterobacteriaceae* and *S aureus* accounted for the highest number of infections in our sample. *Candida* was responsible for 10.1% of the infectious episodes and *Pseudomonas* spp and *Acinetobacter* spp accounted for the majority of nonfermenters (85.0%).

**Table 1:**
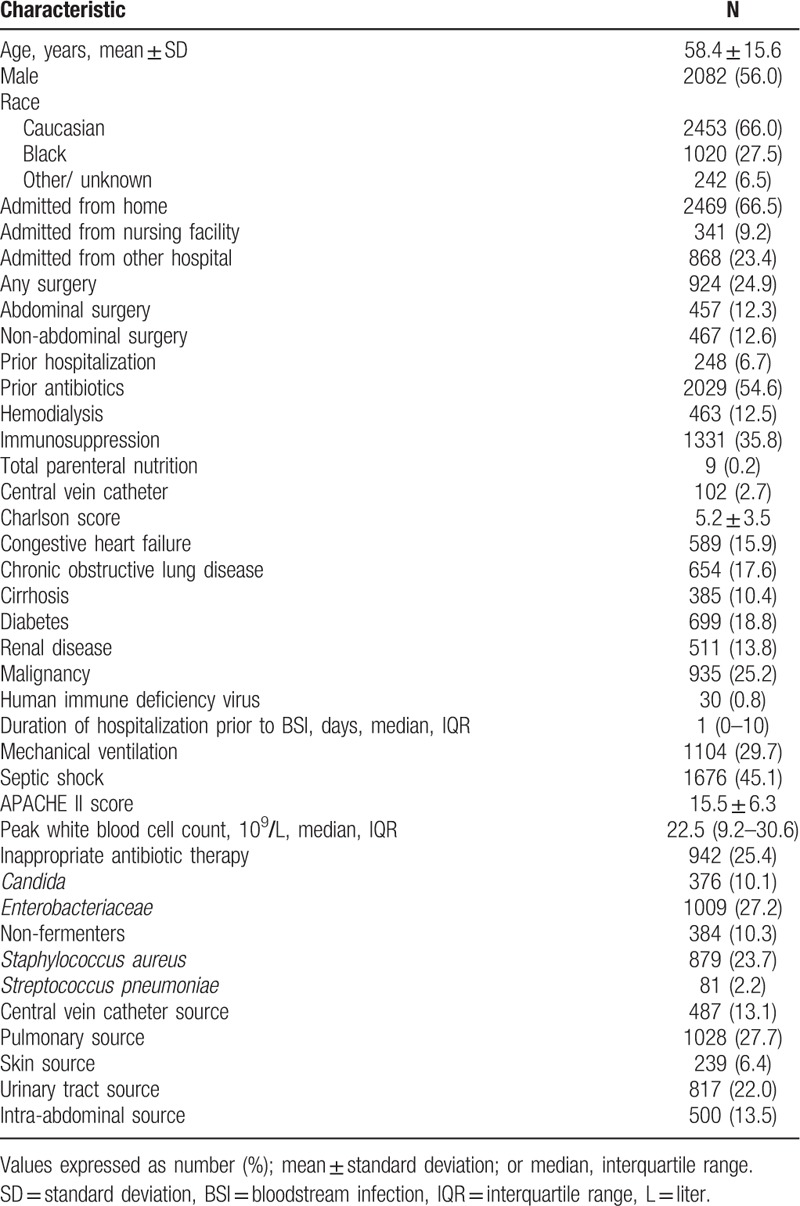
Baseline characteristics for entire cohort.

**Figure 1:**
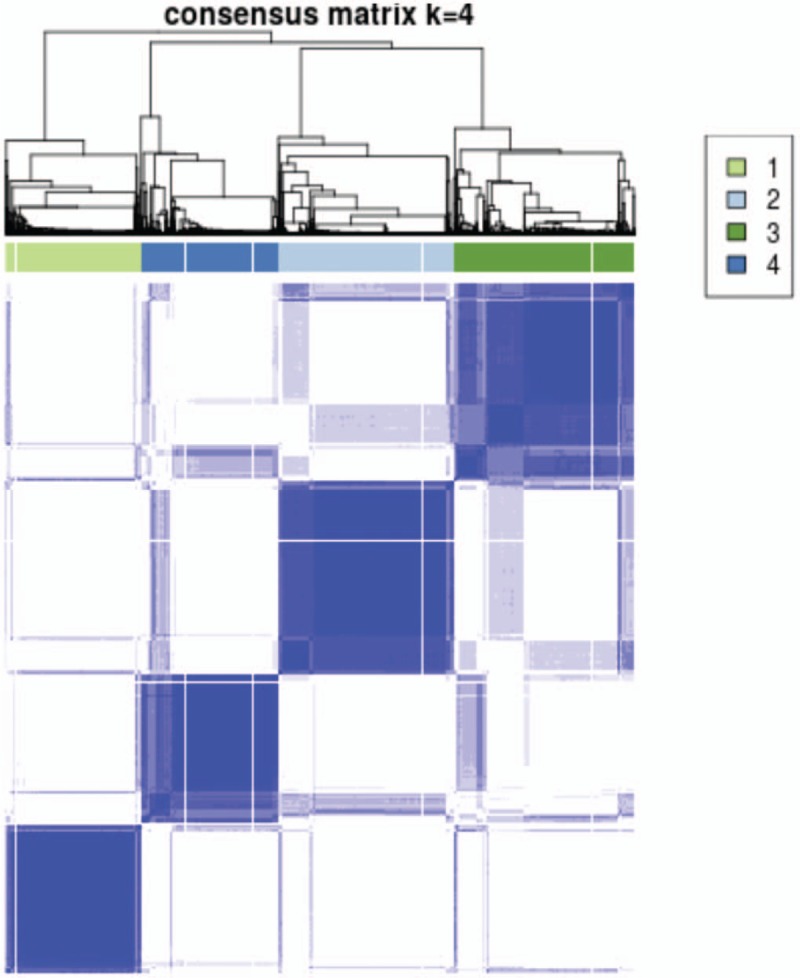
Consensus matrix for 4 clusters (k = 4). The most stable cluster arrangement occurred with formation of 4 clusters with demonstrated block diagonal pattern in the consensus matrix. The dark blue rectangles show the patients assigned to the 4 clusters while the light blue lines represent the unassigned patients.

The most stable cluster arrangement occurred with formation of 4 clusters with demonstrated block diagonal pattern in the consensus matrix (Fig. 1). PAC value was 0.27. This clustering arrangement resulted in an approximately uniform distribution of the population between the four clusters: 800 patients (21.5%), 1037 patients (27.9%), 1068 patients (28.7%), and 810 patients (21.9%) (Table 2).

**Table 2:**
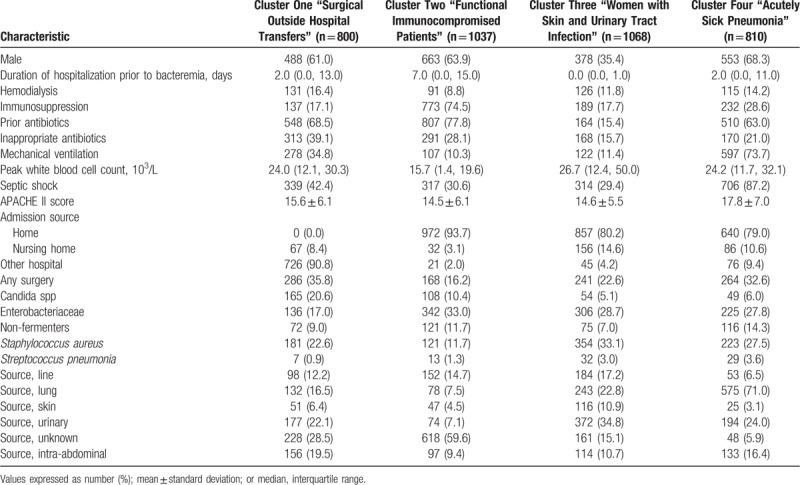
Phenotype summaries for Clusters.

Cluster One called “Surgical Outside Hospital Transfers” was mainly characterized by patients transferred from outside hospitals (90.8%) who had undergone recent surgery and had bacteremia secondary to either a urinary tract or intra-abdominal source. Almost half (43.9%) of all *Candida* infections were grouped in Cluster One.

Cluster Two named “Functional Immunocompromised Patients” was made up primarily of immunocompromised individuals admitted from home with unknown sources of bacteremia, most often secondary to *Enterobacteriaceae* spp. Immunosupression was due to underlying malignancy treated with chemotherapy in almost half of the cluster (Supplementary Table 1). Patients had a significantly longer duration of hospitalization prior to bacteremia compared to the other clusters (7 days vs 2 and 0 days). The administration of prior antibiotics had occurred in 77.8%.

Cluster Three named “Women with Skin and Urinary Tract Infection” was the only cluster dominated by females (64.6%). Even though most individuals within Cluster Three were admitted from home, 45.5% of the total number of nursing home patients aggregated within Cluster Three. The duration of hospitalization prior to BSI was significantly shorter in Cluster Three and a significant proportion of patients had urinary tract infections. Although only 10.9% of the Cluster Three infections were attributed to skin infections, 48.5% of the patients having skin and soft tissue infections grouped in Cluster Three. *Enterobacteriaceae* and *S aureus* accounted for most infections and the patients within Cluster Three were the least likely to receive inappropriate antibiotic therapy. Moreover, patients in Clusters Two and Three appeared to have lower acuity of illness as determined by APACHE II scores and the need for vasopressors or mechanical ventilation.

Cluster Four named “Acutely Sick Pneumonia” was comprised predominantly of critically ill patients as evidenced by the high requirements for vasopressor support and mechanical ventilation, along with higher APACHE II scores. The source of bacteremia was the lung in over 71% of cases and the predominant microbiology varied including *S aureus*, nonfermenters, and *Enterobacteriaceae*. Almost one-third of BSIs attributed to nonfermenting GNB were grouped in Cluster Four along with 35.8% of the BSIs attributed to *S pneumoniae*.

In terms of bacterial species, BSIs caused by *S aureus* distributed to Cluster Three (40%) and Cluster Four (25%), while *Enterobacteriaceae* were divided predominantly into Clusters Two (34%), Three (30%), and Four (22%). Nonfermenting GNB grouped mainly in Clusters Two and Four (31% and 30%). More than half of the pneumonia cases (56%) occurred in Cluster Four, while 37.8% of the catheter-associated bloodstream infections were in Cluster Three. Median white blood cell count was highest for patients in Cluster Three at 26700 cells/L. Cluster One contained 33% of the individuals receiving inappropriate antibiotic administration and Cluster Two contained 31% of these cases. Mortality was greatest for individuals within Cluster Four at 44.6% (Table 3). Cluster One patients were more likely to be discharged to a nursing home (40.1%) while Cluster Two patients were the most likely to be discharged home - 54.2% (Table 3).

**Table 3:**
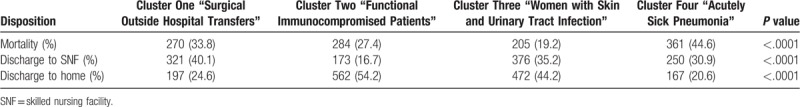
Distribution of mortality and discharge disposition for Clusters.

**Figure 2:**
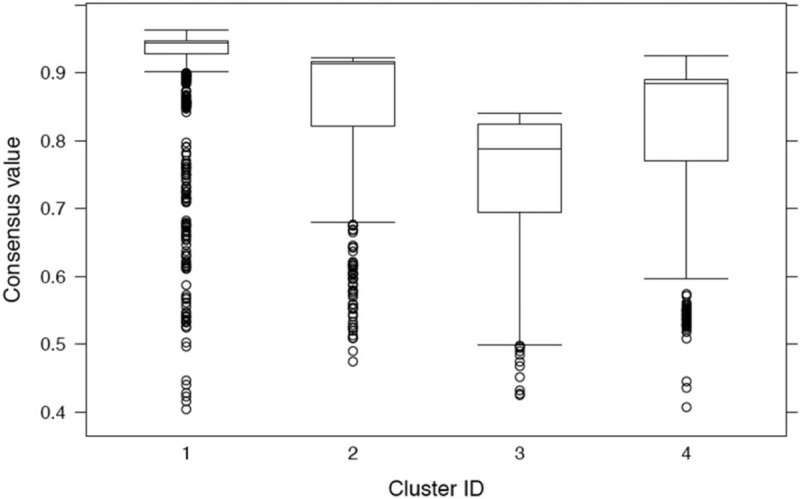
Consensus values across the clusters. Consensus values represent the proportion of times 1 observation (patient) was assigned to the same cluster. For instance, an observation with a consensus value of 93 for cluster one means it was assigned to cluster 1 920 times out of 1000. The Y axis presents the consensus values as box plots with median and interquartile range along with outliers. Cluster One had a consensus value of 0.93, Cluster Two of 0.91, Cluster Three of 0.79, Cluster Four of 0.89.

The number of outliers was small and it was roughly equally distributed across the 4 clusters [Clusters One, 54 (6.72%); Two, 62 (6%); Three, 81 (7.6%); Four, 61 (7.5%)]. The distribution of outcomes was maintained when calculated for the “purified” clusters after excluding the outliers. The distribution of consensus values across the 4 clusters including outliers was high ranging between 0.79 for Cluster Three to 0.93 for Cluster One (Fig. 2).

Given the electronic health records mining and manual data extraction, the missing data were limited to <5%.

## 4 Discussion

We applied clustering to a large database of patients with BSIs and severe sepsis or septic shock and identified four distinct groups with prognostic differentiation. Mortality varied amongst the clusters ranging from a low of 19.2% to a high value of 44.6%. We also found that the clusters segregated patients according to differing dispositions post hospital discharge with Cluster One having the highest discharge rate to skilled nursing facilities. It is also interesting that our groupings did not necessarily aggregate patients only around known and commonly used infectious disease classifiers such as bacterial species or infection source. Our study represents the first analysis employing clustering to construct homogenous groupings of patients with BSIs. The distinctiveness of the identified clusters is supported by their correlation with differing outcomes and discharge dispositions. Moreover, we also identified few outliers and “purified” clusters had similar correlations to outcomes as our initial clustering results also supporting their robustness.

Previous investigations have attempted to identify clinical factors impacting mortality in patients with BSIs. Certain risk factors to include severity of illness, presence of infection with multidrug resistant bacteria, inappropriate initial antibiotic therapy, and comorbid conditions have been identified as independent risk factors of mortality in BSIs and sepsis.^[21–23]^ Interestingly, we found that the cluster with the highest rate of inappropriate initial antibiotic therapy (Cluster One) did not have the greatest mortality. In fact the highest mortality was observed in Cluster Four despite having one of the lower rates of inappropriate initial antibiotic therapy. This suggests that factors other than inappropriate antibiotic therapy may also be important in determining patient outcome. This observation is consistent with our previous results demonstrating that among patients with bacteremic pneumonia, mortality was highest for those with pneumonia attributed to *Pseudomonas aeruginosa* despite inappropriate initial antibiotic therapy being greatest amongst patients infected with antibiotic-resistant *Enterobacteriaceae*.^[2]^ Cluster Four also had the highest rate of infection with nonfermenting Gram-negative bacteria suggesting that underlying virulence of the offending pathogens likely contributed to the higher mortality.^[24]^

The ability to identify cluster–associated outcomes can be useful from many viewpoints. Machine learning techniques such as cluster analysis can be employed to insure that populations are similar relative to the outcome of interest in clinical trials of novel therapies.^[25]^ Similarly, the ability to identify clinically important groupings has potential implications for the management of seriously ill patients including those with BSIs. Machine learning techniques may be able to identify clusters of individuals who are more likely to respond to specific therapies or benefit from different diagnostic approaches. For example, 1 potential clinical application as suggested by our results would be that Cluster One patients might be most likely to benefit from initial broad-spectrum antibiotics or application of rapid microbiologic diagnostics given the higher rate of inappropriate initial antibiotic therapy within this cluster. Grouping methodologies could also allow for improved outcome comparisons between hospitals, especially with increasing requirements for public reporting of such data through systems such as the Severe Sepsis/Septic Shock Early Management Bundle and New York State's Rory's Regulations.^[26,27]^

The strengths of our study are that we had a large sample size to perform clustering, the clusters we obtained seem to make clinical sense and are consistent with previous studies using alternative statistical techniques, and the we were able to assign the majority of the patients to a cluster. There are important limitations of our study that should be noted. First, the data are from a single center so that the groupings may be unique to that population and variables included. Second, consensus clustering can lead to inaccurate numbers of clusters with little discriminatory power. Moreover, cluster analyses may create structured groups even when no structure is present in heterogeneous data sets. However, the correlation and validation of our clusters with pertinent outcomes supports the clinical relevance of the groupings we identified. Given the repeated subsampling, splitting the sample into derivation and validation cohorts was considered unnecessary. Finally, we may have missed entering other clinically important variables and processes of care in our analysis that could have improved the discriminatory ability of the groupings we identified.

New methods are needed to advance the practice of infectious diseases especially in critically ill patients. Machine learning methods such as cluster analysis offers the ability to more efficiently analyze large volumes of data to better understand the underlying risk for acquisition of infectious diseases and transmission pathways, develop targeted interventions, and potentially reduce nosocomial infections and improve patient outcomes.^[12]^ Our results support the potential for machine learning methods to identify more homogenous groupings in infectious diseases that transcend old a priori classifications. These methods may allow new clinical phenotypes to be identified, improve severity staging of complex infectious diseases which currently are rudimentary, and more directly target therapies and diagnostics. An excellent example is the use of newly developed immune checkpoint inhibitors. Clustering patients opens new hypotheses about immune pathways and mediators that may be similar for 2 patients suffering from different infections and microbiology while at the same time dissimilar for 2 patients with the same diagnostic. Clustering analysis will also aid with patient recruitment permitting more generalized entry criteria. With new and expensive or risky treatments entering the field of infectious disease (e.g., monoclonal antibodies, specific pathogen-directed antibiotics, immune stimulatory agents), we need to find the groups of patients that are more likely to get the highest benefit. Medicine is becoming more personalized, yet the available clinical data repositories are highly multidimensional so that finding clinically relevant patterns is more difficult. Our findings suggest that machine learning methods may be part of the solution to this problem.^[28,29]^

## Author contributions

**Conceptualization:** M Cristina Vazquez Guillamet, Scott T Micek, Marin H Kollef.

**Data curation:** M Cristina Vazquez Guillamet, Michael Bernauer, Scott T Micek, Marin H Kollef.

**Formal analysis:** M Cristina Vazquez Guillamet, Michael Bernauer, Scott T Micek, Marin H Kollef.

**Investigation:** M Cristina Vazquez Guillamet, Michael Bernauer, Scott T Micek, Marin H Kollef.

**Methodology:** M Cristina Vazquez Guillamet, Scott T Micek, Marin H Kollef.

**Project administration:** Marin H Kollef.

**Resources:** M Cristina Vazquez Guillamet, Scott T Micek, Marin H Kollef.

**Software:** Michael Bernauer, Marin H Kollef.

**Supervision:** Marin H Kollef.

**Validation:** M Cristina Vazquez Guillamet, Michael Bernauer, Scott T Micek, Marin H Kollef.

**Visualization:** Marin H Kollef.

**Writing – original draft:** Scott T Micek, Marin H Kollef.

**Writing – review & editing:** M Cristina Vazquez Guillamet, Michael Bernauer, Scott T Micek, Marin H Kollef.

## Supplementary Material

Supplemental Digital Content
