# Supplementary material for: Cluster analysis to define distinct clinical phenotypes among septic patients with bloodstream infections
Source: Medicine (Baltimore). 2019 Apr 19;98(16):e15276. doi: 10.1097/MD.0000000000015276 (PMC6494365; doi:10.1097/MD.0000000000015276)
Supplement: Supplemental Digital Content [file medi-98-e15276-s001.doc]

Supplement Table 1. Baseline Characteristics by Cluster

| Characteristic | Cluster 1  “Surgical Outside Hospital Transfers”  (n= 800) | Cluster 2  “Functional Immunocompromised Patients” (n=1037) | Cluster 3  “Women with Skin and Urinary Tract Infection” (n=1068) | Cluster 4  “Acutely Sick Pneumonia”  (n= 810) |
| --- | --- | --- | --- | --- |
| Charlson score, mean ± SD | 4.0 ± 3.1 | 5.5 ±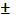3.3 | 5.5 ± 3.6 | 5.7 ± 3.5 |
| CHF | 106 (13.2) | 168 (16.2) | 202 (18.9) | 113 (14.0) |
| COPD | 120 (15.0) | 172 (16.6) | 230 (21.5) | 132 (16.3) |
| Cirrhosis | 64 (8.0) | 141 (13.6) | 112 (10.5) | 68 (8.4) |
| Diabetes | 129 (16.1) | 200 (19.3) | 254 (23.8) | 116 (14.3) |
| Renal disease | 85 (10.6) | 160 (15.4) | 181 (16.9) | 85 (10.5) |
| Malignancy | 103 (12.9) | 498 (48.0) | 231 (21.6) | 103 (12.7) |
| HIV | 3 (0.4) | 16 (1.5) | 3 (0.3) | 8 (1.0) |
| TPN | 4 (0.5) | 2 (0.2) | 0 (0.0) | 3 (0.4) |
| Central line | 42 (5.2) | 23 (2.2) | 30 (2.8) | 7 (0.9) |

Values expressed as number (%) and mean + standard deviation. CHF = congestive heart failure; COPD = chronic obstructive pulmonary disease; HIV = human immune deficiency virus; TPN = total parenteral nutrition; CVC = central vein catheter, SD = standard deviation
